# Oral and Dental Health Status among Adolescents with Limited Access to Dental Care Services in Jeddah

**DOI:** 10.3390/dj6020015

**Published:** 2018-05-17

**Authors:** Salma A. Bahannan, Somaya M. Eltelety, Mona H. Hassan, Suzan S. Ibrahim, Hala A. Amer, Omar A. El Meligy, Khalid A. Al-Johani, Rayyan A. Kayal, Abeer A. Mokeem, Akram F. Qutob, Abdulghani I. Mira

**Affiliations:** 1 Oral and Maxillofacial Prosthodontics Department, Faculty of Dentistry, King Abdulaziz University, Jeddah 21589, Saudi Arabia; 2 Dental Public Health Department, Faculty of Dentistry, Al Mansoura and King Abdulaziz Universities, Jeddah 21589, Saudi Arabia; saltalety@kau.edu.sa; 3 Biostatistics Department, High Institute of Public Health, Alexandria University and Dental Public Health Department, Faculty of Dentistry, King Abdulaziz University, Jeddah 21589, Saudi Arabia; monaha59@hotmail.com; 4 Oral Diagnostic Sciences Department, Faculty of Dentistry, Ain Shams and King Abdulaziz Universities, Jeddah 21589, Saudi Arabia; suzan_ibrahim2000@yahoo.com; 5 Dental Public Health Department, Faculty of Dentistry, Alexandria and King Abdulaziz Universities, Jeddah 21589, Saudi Arabia; halaamerdr@gmail.com; 6 Pediatric Dentistry Department, Faculty of Dentistry, King Abdulaziz and Alexandria Universities, Jeddah 21589, Saudi Arabia; omeligy@kau.edu.sa; 7 Oral Diagnostic Sciences Department, Faculty of Dentistry, King Abdulaziz University, Jeddah 21589, Saudi Arabia; kauoralmed@gmail.com; 8 Periodontics Department, Faculty of Dentistry, King Abdulaziz University, Jeddah 21589, Saudi Arabia; rkayal@kau.edu.sa; 9 Endodontics Department, Faculty of Dentistry, King Abdulaziz University, Jeddah 21589, Saudi Arabia; aasaleh@kau.edu.sa; 10 Dental Public Health Department, Faculty of Dentistry, King Abdulaziz University, Jeddah 21589, Saudi Arabia; aqutob@kau.edu.sa; 11 Conservative Dentistry Department, Faculty of Dentistry, King Abdulaziz University, Jeddah 21589, Saudi Arabia; amira@kau.edu.sa

**Keywords:** oral health, caries, student, habits, risk factors, limited access, oral health survey

## Abstract

The purpose of this study was to assess the prevalence and associated factors of dental caries and periodontal diseases among 14–19-year-old schoolchildren with limited access to dental care services. A cross sectional study design was conducted during field visits to seven governmental schools in Al-Khomrah district, South Jeddah, over the period from September 2015 to May 2016. Clinical examinations and administered questionnaires were carried out in mobile dental clinics. The dentists carried out oral examinations using the dental caries index (DMFT), the simplified oral hygiene index (OHI-S), and the community periodontal index for treatment needs (CPITN). Statistical analyses were performed using SPSS 20. A total of 734 schoolchildren were examined. The prevalence of decayed teeth was 79.7% and was significantly higher among boys (88.9%) than girls (69.0%). About 11% of students had missing teeth, with a significantly higher figure among females than males (15.9% versus 7.3%); 19.8% of students had filled teeth. Moreover, a DMFT of seven or more was significantly more prevalent among males (43.3%) than females (26.8%), while the percentage of females with sound teeth was significantly higher than for males (20.4% and 9.6% respectively). The CPITN revealed 0, 1 and 2 scores among 14.6%, 78.2%, and 41.6% respectively. Males had a significantly higher percentage of healthy periodontal condition (23.8%) than females (3.8%). Dental caries prevalence was moderate to high, calculus and gingival bleeding were widespread among schoolchildren, and were more prevalent among students with low socioeconomic status.

## 1. Introduction

Oral hygiene is essential to general health and quality of life. The common oral diseases are caries, periodontal disease, erosion, abfraction lesions and oral cancer [1]. Oral health is a state of being in which an individual is free from mouth and facial pain, oral and throat cancer, oral infection and sores, periodontal disease, tooth decay, tooth loss, and other diseases and disorders that limit an individual’s capacity to bite, chew, smile, speak, as well as his or her psychosocial wellbeing. Risk factors for oral diseases include an unhealthy diet, tobacco use, harmful alcohol use, poor oral hygiene, and social determinants [1]. The United States Department of Health and Human Services (HHS) reported in 2014 that dental caries is the most common chronic disease. It is five times more common than asthma, and seven times more common than seasonal allergies [2]. In a recent review, it was documented that the prevalence of caries is high across Saudi Arabia: about 70% in children’s permanent teeth, with a mean DMFT score of 3.5 [3]. Al Dosari et al., reported that the prevalence of caries in the dentition of Saudi children aged 15–18-year old ranged from 59–80%, depending on the fluoride level of the area, and the mean DMFT scores was 2.24–4.08 [4].

It is clearly known that the prevalence of oral disease varies according to geographical region and accessibility of oral health services. With contemporary understanding of social sciences, it has been acknowledged that oral health is influenced by many social and environmental factors. One such factor is accessibility to oral health care services. Limited access to oral health care can be accounted for on the level of the patient, community, inadequate insurance coverage, and a limited supply of oral health care providers. While the delivery of quality care is important, access to oral health is an important factor in maintaining oral health. Individual knowledge, the perceptions of one’s need for oral health care, financial concerns, and cultural preferences can influence patients’ pursuit for oral health care [5]. While the delivery of quality care is important, access to oral health is an equally important determinant of oral health [1].

Many studies have reported that oral diseases are significantly more prevalent among poor and disadvantaged population groups [1,6,7,8,9]. It has been reported that oral diseases have a negative impact on the quality of life, in both developing and developed countries [10,11]. In addition, they restrict activities in school and home, and present challenges for maintaining self-esteem and attentiveness to learning [12].

Although Saudi Arabia has a considerable number of dental colleges, there is glaring disparity in the distribution of these institutions. A geographic imbalance in the availability of oral health care services affect the dentist–population ratio. Most of the Saudi population who reside in rural areas have limited access to oral health care facilities [3].

The objective of the current study was to assess the prevalence and associated factors of caries and periodontal diseases among school students with limited access to dental care services in the Al-Khomrah district in South Jeddah, Saudi Arabia.

## 2. Methods

This was a cross sectional study design that took place in Al-Khomrah district in South Jeddah, Saudi Arabia, during the period from September 2015 to May 2016. The target population was chosen based on its low socioeconomic classification among the Districts of South Jeddah. This classification was obtained from the Ministry of Social Affairs.

Initial site visits then took place to check the logistics of the field visits, and to obtain verbal consent from the principals of Al-Khomrah intermediate and secondary schools. Ethical approval was then obtained from the Ethics Committee of the Faculty of Dentistry at King Abdulaziz University (KAU), Jeddah (REC-FD # 006-15). This was followed by contacting all concerned authorities (the Ministry of Education, the local municipality, and the District’s Governor’s Office), in order to obtain official approval and to facilitate the field visits.

Clinical examination forms and administered questionnaires were developed and tested during the period of examiner calibration at the Faculty of Dentistry, KAU. Questionnaire items were developed after taking input from different clinical expert focus groups, in order to establish validity. These items reflected factors related to oral health status, such as social history, and barriers to care. Designated examiners went through calibration sessions to standardize inter- and intra-examiner reliability. Each examiner conducted five patient examinations to assess dental caries, oral hygiene status, and the gingival condition using the DMFT [13], OHI-S [14] and CPITN [13] indices respectively. Calibration sessions were repeated until the level of consistency reached 85% or higher.

Prior to the start of the field visits, parental informed consent forms were distributed by the participating school. The latter collected signed forms and prepared a list of consensual students in preparation for the field visits. These consents included permission to perform the administered questionnaire, a clinical examination, and provision of basic dental treatments (preventive, restorative, and surgical). Participants requiring advanced dental treatments were given referral slips to continue their treatment at the KAU Faculty of Dentistry clinics, or at the nearest local Ministry of Health primary health care center. At the end of each visit, students were given a summary report of the treatment provided, to inform their parents or care givers. The field visits were undertaken at seven schools: four intermediate schools, and three secondary schools.

The dental team consisted of six general dentists and four dental assistants. The mobile dental clinic car was parked at each school until all participating schoolchildren have been examined. The examiners conducted clinical examinations and provided basic dental treatment in the two mobile clinics (which were stationed in the carpark), and in the first aid rooms of the schools using two portable dental units (ProCart II, South Pointe Surgical Supply, Inc., Coral Springs, FL, USA). Infection control and sterilization standards were employed at all examination sites to ensure the safety of participants.

Data was entered after removing all participants’ identifiers to protect confidentiality, and group analyses were performed using IBM SPSS 20, (IBM, Armonk, NY, USA). Descriptive statistics and odds ratios were calculated and presented, as seen in the results section. All tests were two-sided, and the 0.05 level was used to indicate statistical significance.

## 3. Results

Demographic characteristics of students by gender is shown in Table 1. The current study included 734 students (53.8% males and 46.2% females), with a mean age of 16.02 ± 1.61 years, with a similar mean age of males and females. The distribution of students according to the education level of their fathers and mothers showed a similar distribution for both boys and girls. A considerable percentage of students’ parents had less than a secondary level of education (in males and females 79.1% and 69.1% respectively). Regarding parents working status, 73.4% of fathers were working, whereas only 11.3% of mothers were working. Considering citizenship, 37 (5%) were non-Saudi, while 697 (95%) were Saudi. Regarding residence, 89 (12.2%) reported rented residence, while 640 (87.8%) reported owned residence. Non-Saudi females reported a significantly higher rate of residence in rented houses than that of males.

As shown in Table 2, males and females are significantly different in both oral hygiene status and habits. The percentage of females who brush their teeth twice daily was significantly higher than that of males (57.8% and 14.9% respectively), while the rate of males who did not brush their teeth, or who brush irregularly, was significantly higher compared to that of females (59.7% versus 19.2% respectively). On the other hand, males use Miswak significantly more than females (32.7% compared to 19.5% respectively). Miswak is a stem or root of the plant Salvadora persica; it becomes brushlike after treatment and is suitable for oral hygiene. The basic technique employed for removing plaque mechanically is similar to that of the toothbrush, i.e., vertical and horizontal brushing [10]. About 9% of students were smokers, without significant difference between males and females. Considering the oral hygiene index (soft deposit), significantly more males had good oral hygiene than females (33.7% and 13.6% respectively), while the percentage of fair oral hygiene was higher among females (71.4%) compared to males (47.3%).

The prevalence of decayed permanent teeth (Table 3) was 79.7% and was significantly higher among boys (88.9%) than girls (69.0%). About 11% of students had missing teeth; this rate was significantly higher among females (15.9% versus 7.3%); 19.8% of students had filled teeth, with a significantly higher rate among females (13.9% and 26.5% respectively).

The prevalence of decayed teeth revealed a decreasing significant trend by age, from 83.7% among children aged 14 ≤ 16 years, down to 69.8% for 18–19 years. A reverse pattern could be observed for missing teeth, where the percentage increased from 7.4% at age 14 ≤ 16 years, up to 16% at the age of 18–19 years. The percentage of filled teeth did not show a statistically significant trend by age.

DMFTs of 7 or more (Table 4) were significantly higher among males (43.3%) than females (26.8%). Moreover, the percentage of females with sound teeth was significantly higher than that of males (20.4% and 9.6% respectively). Non-Saudi students showed a significantly higher prevalence of a DMFT index of 7 or more (59.5%) than Saudi students (34.4%). Students who brush their teeth twice daily showed the lowest prevalence of severe dental caries (29.8%), compared to those who never brush their teeth (44.4%). The percentage of students who have severe dental caries was higher among those who visit dentists regularly every six months (43.6%) or every 12 months (72.7%) than those who did not visit dentists (29.1%).

Students with good oral hygiene (soft deposits) had a significantly higher percentage of sound teeth (14%) and a lower percentage of DMFT of 7 or more (35.8%) than those with poor oral hygiene (8.7% and 49.2% respectively). No significant relationship could be detected between DMFT categories and other socio-demographic variables.

The CPITN is presented in Table 5. Males have a significantly higher percentage of healthy periodontal conditions (23.8%) than females (3.8%). While the percentage with bleeding was higher among females (92% versus 66.3% respectively), and 53.1 versus 31.6% respectively with calculus. The youngest age category was more likely to have healthy gingiva in contrast to the 18–19 age category (OR 2.0, 95% CI 1.2 to 3.4), while the latter age category are 1.5 times more prone to calculus deposits, in contrast to the 14 ≤ 16 years age category.

Non- and infrequent-users of tooth brushes were less likely to have bleeding, while non-users of Miswak were more likely to have calculus. Students with good oral hygiene were more likely to have sound teeth regarding CPITN, while those with fair or poor oral hygiene were more likely to have bleeding or calculus.

Regarding standardization and measurement processes for oral and dental health, a comparison among the results of the examiners and the analysis was done to determine the level of consistency between them. An acceptable level of consistency was 85% or more [13]. Inter- and intra-examiner agreement ranged from 0.75 to 0.866, and 0.933 to 0.989 respectively.

## 4. Discussion

Our data shows a high prevalence of dental caries, calculus, and gingival bleeding among 14–19-year-old schoolchildren from low socioeconomic status background in the Al-Khomrah district, South Jeddah, Saudi Arabia. This data may be of importance in the evaluation of past and future planning of oral health prevention and treatment programs targeting schoolchildren. This calls for early preventive strategies and treatment services. We recommend the incorporation of oral health education in school curricula to help in improving the oral health status of schoolchildren with limited access to oral health care services. 

With a contemporary understanding of social sciences, it has been acknowledged that oral health is influenced by many social and environmental factors. One such factor is accessibility to oral health care services. Individual knowledge, the perceptions of one’s need for oral health care, financial concerns, and cultural preferences can influence the priority that patients place upon oral health care [5].

The impact of such limited access to health care services is of even greater consequence on strata of the population, such as children. They depend entirely on their parents to utilize health care services [5]. Hence, this study aimed to assess the oral health status of intermediate and secondary schoolchildren with limited access to oral health care services in the districts of Al-Khomrah in South Jeddah, in order to raise the level of health awareness and to promote good habits for oral health. In addition, early treatment and following preventive methods can decrease dental caries and tooth loss [11].

As for behavior, tooth brushing was the most common method used for cleaning teeth, followed by Miswak. The least common method was the dental floss. This agrees with previous studies [15,16,17]. This might suggest a lack of awareness and understanding of the procedure, and its value in preventing oral disease among the subjects.

Because of the scientific merit of using Miswak and the special importance of the cultural and religious beliefs deeply rooted in, and affecting the behaviors of, the Saudi population, the right method of using a Miswak as a cleaning technique to achieve maximum benefits should be stressed through various interventions [16]. Regarding the frequency of brushing, flossing, and use of Miswak in relation to gender, it was found that females used brushing and flossing more than males. The significant difference (*p* < 0.001) was attributed to a higher concern regarding personal hygiene and health care among females [15,18]. However, males used Miswak more than females; the difference was statistically significant (*p* < 0.001). This result could be justified by cultural beliefs among Saudi communities [16]. The result indicates that improvements in knowledge of the role of dental floss are needed; this agrees with other studies [15,16,17,18].

In relation to dental visits, the majority (45.8%) of students only visited their dentist when needed, followed by those who never visited a dentist (44.6%); a minority visited a dentist every 6 to 12 months (6.8%). This agrees with previous studies [19,20]. This may be due to the lack of oral health knowledge among these students. Delay in seeking dental care could be also attributed to other factors, like parental beliefs and practices, lack of economic resources, and the accessibility of dental services [21]. Such a negative attitude towards visiting a dentist leads to an inability to avail sound advice on preventive oral health practices and counseling, a high prevalence of dental caries, and delayed recognition and management of carious teeth [20]. In the current study, there was a significant difference in the frequency of dental visits by gender (*p* < 0.001). Females showed more frequent visits than males.

Four hundred and twenty-nine students (58.4%) had fair oral hygiene (soft deposits), which is less than reported in Kuwait (67%) [22] and in Nigeria (72%) [23]. There were significant sex differences in oral hygiene status, where fair status was observed to be higher in females than males. It was reported that females pay more attention to their personal hygiene, and tend to practice better oral hygiene than males, because of their greater social awareness and grooming habits [24].

Despite incredible scientific advances and the fact that caries are preventable, the disease continues to be a major public health problem. The World Health Organization (WHO) has ranked it as number three among all chronic non-communicable diseases that require worldwide attention for prevention and treatment [1]. Moreover, decayed teeth are particularly harmful to children’s growth and development and can severely jeopardize their health [25]. Therefore, reliable estimations of its prevalence will play an important role in improving oral health.

This study showed that only 14.6% were free from caries, which indicates a high prevalence of caries (85.6%) and DMFT (>7). This is in accordance with findings of previous studies in Saudi Arabia [20,26,27]. This rate was higher than Riyadh (70%) [4], or Iran, where the prevalence of dental caries was 75.5% [28]; in Istanbul, the rate is 80% [29].

In a previous study by Al-Ansari, [30] it was found that the increased prevalence of caries in Saudi communities was related to tremendous growth in population coupled with social changes, unhealthy oral health behaviors and practices, inadequate access to oral health care, particularly in remote areas, non-availability of fluoridated water, and paucity of clinical and population-based research.

Dental decay was the major component of the DMFT scores in this study, as evidenced by the high prevalence of decayed teeth (79.7%), which agrees with studies in the Riyadh and Qasim regions. Several studies of younger children in Riyadh have also reported decay as the major component of DMFT [26,31,32]. Such a large proportion of untreated caries indicates the extent of restorative needs among the studied population; consequently, a considerable effort will be required to provide restorative services to them.

Regarding gender, it has been found that boys showed a significantly higher prevalence of caries and missing teeth than girls. This may be because Saudi girls are more concerned about their oral health than Saudi boys. This agrees with other studies in Riyadh [33], while the prevalence of filled teeth was higher in females, suggesting that girls showed a preference for receiving dental treatment compared to boys.

The result of this study showed a significant relationship between DMFT and the frequency of oral hygiene practices: students who brushed regularly (twice/day) had the lowest prevalence of severe dental caries (29.8%), in comparison to those who never brush their teeth (44.4%); this is consistent with the findings of Dummer et al. [24].

Moreover, severe dental caries were more common among those who visited a dentist regularly (every 6 or every 12 months) than those who did not. This agrees with a study in China [34]. The higher DMFT score can be attributed to the dental visit patterns of the studied group. Most of those in this group visited dentists only when they had a problem that required treatment. Studies have also shown that there is a relationship between dental caries and socioeconomic status. In addition, parental income level, educational level, employment status, and other socioeconomic factors have a considerable impact on the prevalence of dental caries [35].

The results of the current study showed that children with the highest prevalence of sound teeth (16.7%) were those having parents with higher levels education, and those who owned a house (15.2%), factors which could reflect a high socioeconomic status. The high prevalence of caries among individuals from lower social class may be linked to the increasing availability of cheap sugar-rich products, coupled with low income and poor access to health services and health education. The low prevalence of caries in members of high social classes may be attributed to increased oral health care awareness among and access to dental care at an earlier age [36].

Regarding the periodontal status, the current study revealed that healthy gingiva was found only in 14.6% of the studied group. Calculus was the most frequently observed condition (78%), while gingivitis was less common (41.6%). This finding agrees with a study by Hessari et al. [37].

Regarding gender, the rates of individuals with bleeding and calculus were higher among females than males. This agrees with the findings of other studies that recorded better gingival health among girls than boys, because the females give more importance to aesthetics and are more inclined to brush and seek dental care regularly [37,38].

This study showed that both socioeconomic background and the utilization of dental services had no effect on periodontal health status, which indicated that some other factors may be at play. This result agrees with that of a comparable study undertaken in Hong Kong [39]. It was also found that students who had good oral hygiene had more favorable periodontal condition, since dental plaque plays an important role in the development of gingival inflammation [40]. More promotion of oral health, and community-based activities, are needed among adolescents.

There are some limitations to this study that should be considered. First, as a cross sectional study, it cannot provide proof for causality, especially in the absence of a control group with better access to dental care services. Second, the sample size was small, and it was conducted in a single, low socioeconomic district. Therefore, further studies are recommended which would include more districts in different areas in Saudi Arabia.

## 5. Conclusions

Dental caries prevalence was moderate to high, and calculus and gingival bleeding were widespread among Al-Khomrah schoolchildren and were more prevalent among students of low socioeconomic status. Furthermore, males had a significantly higher prevalence of caries and missing teeth than females. More oral health promotion, and community-based activities are needed among the surveyed adolescents.

## Figures and Tables

**Table 1:** Demographic characteristics of students by gender.

Characteristics		No. Examined 734		Males 395 (53.8%)		Females 339 (46.2%)		X^2^	*p*
Age (Years)	14–	326	44.4	176	44.6	150	44.2	0.57	0.774
	16–	239	32.6	132	33.4	107	31.6		
	18–19	169	23.0	87	22.0	82	24.2		
	Mean (SD)	16.02	(1.61)	16.02	(1.59)	16.02	(1.64)	t = 0.02	0.983
Father’s Education ◈	No education	105	15.2	61	16.1	44	14.1	2.97	0.563
	Primary school	158	22.8	93	24.5	65	20.8		
	Intermediate school	146	21.1	73	19.2	73	23.3		
	Secondary school	186	26.8	100	26.3	86	27.5		
	University or higher	98	14.1	53	13.9	45	14.4		
Mother’s education ⁂	No education	179	25.3	111	29.3	68	20.7	9.42	0.052
	Primary school	163	23.1	78	20.6	85	25.9		
	Intermediate school	146	20.7	70	18.5	76	23.2		
	Secondary school	149	21.1	83	21.9	66	20.1		
	University or higher	70	9.9	37	9.8	33	10.1		
Fathers’ work	Yes	519	73.4	278	72.8	241	74.2	0.17	0.679
	No	188	26.6	104	27.2	84	25.8		
Mothers’ work	Yes	81	11.3	45	11.7	36	10.9	0.12	0.732
	No	635	88.7	340	88.3	295	89.1		
Citizenship	Saudi	697	95.0	381	96.5	316	93.2	4.00 *	0.045
	Non-Saudi	37	5.0	14	3.5	23	6.8		
Residence	Owned	640	87.8	352	90.0	288	85.2		
	Rented	89	12.2	39	10.0	50	14.8	3.93 *	0.048

◈ 16 males and 11 females with missing mothers’ education; ⁂ 15 males and 26 females with missing fathers’ education; * *p* < 0.05 (Significant).

**Table 2:** Oral hygiene status and habits of students by gender.

Characteristics		No. Examined 734		Males 395 53.8%		Females 339 46.2%		X^2^	*p*
Daily tooth brushing	Twice	255	34.7	59	14.9 ^A^	196	57.8 ^A^	191.07 *	<0.001
	Once	178	24.3	100	25.3	78	23.0		
	Not frequently	184	25.1	125	31.6 ^B^	59	17.4 ^B^		
	Never	117	15.9	111	28.1 ^C^	6	1.8 ^C^		
Miswak	Yes	195	26.6	129	32.7	66	19.5	16.27 *	<0.001
	No	539	73.4	266	67.3	273	80.5		
Tooth flossing	Yes	43	5.9	19	4.8	24	7.1	1.70	0.192
	No	691	94.1	376	95.2	315	92.9		
Dental visits	Every 6 months	39	5.3	27	6.8 ^A^	12	3.5 ^A^	32.12 *	<0.001
	Every 12 months	11	1.5	8	2.0	3	0.9		
	Irregular	21	2.9	7	1.8	14	4.1		
	When needed	336	45.8	148	37.5 ^B^	188	55.5 ^B^		
	Never	327	44.6	205	51.9 ^C^	122	36.0 ^C^		
Smoking	No	669	91.1	354	89.6	315	92.9	2.46	0.117
	Yes	65	8.9	41	10.4	24	7.1		
OHI-S (Soft deposit)	<1 (Good)	179	24.4	133	33.7 ^A^	46	13.6 ^A^	49.93	<0.001
	1– (Fair)	429	58.4	187	47.3 ^B^	242	71.4 ^B^		
	2+ (Poor)	126	17.2	75	19.0	51	15.0		

Percent with common superscripts for the same variable are significantly different; * *p* < 0.05 (Significant).

**Table 3:** Dental caries by age and gender.

		No. Examined	Caries prevalence						Median DMFT	95% CI	
			D		M		F				
			No.	%	No.	%	No.	%			
Overall		734	585	79.7	83	11.3	145	19.8	4.0	4.0	5.0
Gender	Males	395	351	88.9	29	7.3	55	13.9	6.0	5.0	6.0
	Females	339	234	69.0	54	15.9	90	26.5	3.0	3.0	4.0
	Test (*p*)		X^2^ = 44.36 * (<0.001)		X^2^ = 13.41 * (<0.001)		X^2^ = 18.34 * (<0.001)		Mann-Whitney Z = 50.75 * (<0.001)		
Age (Years)	14–	326	273	83.7 _A_	24	7.4 _A_	58	17.8	4.0	4.0	5.0
	16–	239	194	81.2 _A_	32	13.4 _AB_	55	23.0	5.0	4.0	6.0
	18–19	169	118	69.8 _B_	27	16.0 _B_	32	18.9	4.0	3.0	5.0
	Test (*p*)		X^2^ = 13.8 * (0.001)		X^2^ = 9.77 * (0.008)		X^2^ = 2.47 (0.292)		Kruskal-Wallis X^2^ = 3.32 (0.190)		

Groups with different subscripts in the same column are significantly different; * *p* < 0.05 (Significant).

**Table 4:** Caries prevalence by students’ characteristics.

		Examined	DMFT (%)				X^2^	*p*
			0	1–3	4–6	7+		
Overall		734	14.6	26.4	23.3	35.7		
Gender	Males	395	9.6	20.8	26.3	43.3	42.03 *	<0.001
	Females	339	20.4	33.0	19.8	26.8		
Age (Years)	14–	326	12.0	26.4	23.6	38.0	11.41	0.076
	16–	239	13.8	25.5	27.2	33.5		
	18–19	169	20.7	27.8	17.2	34.3		
Father’s Education ◈	No education	105	16.2	25.7	23.8	34.3	10.50	0.572
	Primary school	158	13.3	20.9	23.4	42.4		
	Intermediate school	146	10.3	30.1	24.0	35.6		
	Secondary school	186	16.7	25.3	27.4	30.6		
	University or higher	98	16.3	28.6	21.4	33.7		
Mother’s Education ⁂	No education	179	14.5	25.1	23.5	36.9	9.57	0.654
	Primary school	163	14.1	26.4	23.3	36.2		
	Intermediate school	146	16.4	23.3	26.7	33.6		
	Secondary school	149	16.8	31.5	18.1	33.6		
	University or higher	70	10.0	25.7	32.9	31.4		
Fathers’ work	Yes	519	13.9	25.0	23.9	37.2	1.82	0.611
	No	188	16.0	28.2	23.4	32.4		
Mothers’ work	Yes	81	8.6	30.9	24.7	35.8	2.92	0.405
	No	635	15.3	25.8	23.5	35.4		
Citizenship	Saudi	697	15.1	27.0	23.5	34.4	10.27 *	0.016
	Non-Saudi	37	5.4	16.2	18.9	59.5		
Residence	Owned	640	15.2	27.2	23.1	34.5	3.38	0.337
	Rented	89	11.2	22.5	22.5	43.8		
Daily tooth brushing	Twice	178	18.0	33.3	18.8	29.8	34.64 *	<0.001
	Once	255	16.9	25.3	17.4	40.4		
	Not frequently	184	12.5	21.2	32.6	33.7		
	Never	117	6.8	21.4	27.4	44.4		
Miswak	Yes	195	14.9	22.1	27.7	35.4	4.15	0.246
	No	539	14.5	28.0	21.7	35.8		
Tooth flossing	Yes	43	11.6	23.3	30.2	34.9	1.40	0.706
	No	691	14.8	26.6	22.9	35.7		
Dental visits	Every 6 months	39	5.1	23.1	28.2	43.6	*	MCP = 0.002
	Every 12 months	11	0.0	9.1	18.2	72.7		
	Irregular	21	14.3	14.3	19.0	52.4		
	When needed	336	10.4	28.3	22.3	39.0		
	Never	327	20.5	26.3	24.2	29.1		
Smoking	No	669	14.6	25.6	23.9	35.9	3.50	0.321
	Yes	65	13.8	35.4	16.9	33.8		
OHI-S (Soft deposit)	<1 (Good)	179	14.0	21.2	29.1	35.8	21.09 *	0.002
	1– (Fair)	429	16.6	30.1	21.7	31.7		
	2+ (Poor)	126	8.7	21.4	20.6	49.2		

◈ 16 males and 11 females with missing mothers’ education; ⁂ 15 males and 26 females with missing fathers’ education; MCP = Monte Carlo exact P; * *p* < 0.05 (Significant).

**Table 5:** Community periodontal index for treatment needs by students’ characteristics.

		Examined	% Healthy	OR (95% CI)	% with Bleeding	OR (95% CI)	% with Calculus	OR (95% CI)
Overall		734	14.6		78.2		41.6	
Gender	Males	395	23.8	7.8 * (4.3–14.3)	66.3	1.0	31.6	1.0
	Females	339	3.8	1.0	92.0	5.9 * (3.8–9.2)	53.1	2.4 * (1.8–3.3)
Age (Years)	14–	326	11.0	2.0 * (1.2–3.4)	81.0	1.0	39.3	1.0
	16–	239	15.5	1.4 (0.8–2.3)	81.2	1.0 (0.7–1.6)	39.3	1.0 (0.7–1.4)
	18–19	169	20.1	1.0	68.6	0.5 (0.3–0.8)	49.1	1.5 * (1.0–2.2)
Father’s Education ◈	No education	105	14.3	1.0	83.8	1.9 (0.9–3.7)	37.1	0.8 (0.4–1.4)
	Primary school	158	15.2	0.9 (0.5–1.9)	70.9	0.9 (0.5–1.5)	44.3	1.1 (0.6–1.8)
	Intermediate school	146	11.0	1.4 (0.6–2.9)	82.9	1.7 (0.9–3.3)	43.8	1.0 (0.6–1.7)
	Secondary school	186	14.5	1.0 (0.5–1.9)	79.6	1.4 (0.8–2.5)	39.2	0.9 (0.5–1.4)
	University or higher	98	20.4	0.7 (0.3–1.4)	73.5	1.0	42.9	1.0
Mother’s Education ⁂	No education	179	18.4	1.0	72.1	0.9 (0.5–1.7)	38.5	0.9 (0.5–1.7)
	Primary school	163	13.5	1.0 (0.5–2.1)	81.0	1.5 (0.8–2.9)	41.7	1.1 (0.6–1.9)
	Intermediate school	146	8.9	1.5 (0.7–3.1)	86.3	2.2 * (1.1–4.5)	42.5	1.1 (0.6–2.0)
	Secondary school	149	15.4	2.3 * (1.0–5.3)	75.8	1.1 (0.6–2.1)	43.0	1.1 (0.6–2.0)
	University or higher	70	18.6	1.2 (0.6–2.6)	74.3	1.0	40.0	1.0
Fathers’ work	Yes	519	14.5	1.0 (0.6–1.5)	78.0	1.0	41.8	1.0
	No	188	14.9	1.0	78.7	1.0 (0.7–1.6)	40.4	0.9 (0.7–1.3)
Mothers’ work	Yes	81	17.3	1.3 (0.7–2.3)	72.8	1.0	46.9	1.0
	No	635	14.2	1.0	79.1	1.4 (0.5–1.2)	40.5	0.8 (0.5–1.2)
Citizen-ship	Saudi	697	14.5	0.9 (0.4–2.2)	78.8	1.0	40.9	1.0
	Non–Saudi	37	16.2	1.0	67.6	0.6 (0.3–1.1)	54.1	1.7 (0.9–3.3)
Residence	Owned	640	14.1	0.7 (0.4–1.2)	80.2	1.0	39.5	1.0
	Rented	89	19.1	1.0	66.3	0.5 * (0.3–0.8)	52.8	1.7 * (1.1–2.7)
Daily tooth brushing	Twice	255	11.4	0.7 (0.4–1.2)	83.5	1.0	42.7	1.0
	Once	178	12.9	0.8 (0.4–1.5)	79.8	0.8 (0.5–1.3)	42.7	1.0 (0.7–1.5)
	Not frequently	184	19.6	1.3 (0.7–2.3)	73.9	0.6 * (0.4–0.9)	39.1	0.9 (0.6–1.3)
	Never	117	16.2	1.0	70.9	0.5 * (0.3–0.8)	41.0	0.9 (0.6–1.5)
Miswak	Yes	195	16.4	1.2 (0.8–1.9)	76.4	1.0	36.4	1.0
	No	539	13.9	1.0	78.8	1.2 (0.8–1.7)	43.4	1.3 * (1.0–1.9)
Tooth flossing	Yes	43	9.3	0.6 (0.2–1.7)	79.1	1.0	44.2	1.0
	No	691	14.9	1.0	78.1	0.9 (0.4–2.0)	41.4	0.9 (0.5–1.7)
Dental visits	Every 6 months	39	15.4	1.1 (0.4–2.8)	71.8	1.0	43.6	1.0
	Every 12 months	11	9.1	2.0 (0.3–16.1)	90.9	3.9 (0.4–34.4)	18.2	0.3 (0.1–1.5)
	Irregular	21	9.5	1.9 (0.4–8.5)	81.0	1.7 (0.5–6.1)	42.9	1.0 (0.3–2.8)
	When needed	336	12.8	1.4 (0.9–2.1)	81.0	1.7 (0.8–3.5)	42.9	1.0 0.5–1.9)
	Never	327	16.8	1.0	75.5	1.2 (0.6–2.5)	40.7	0.9 (0.5–1.7)
Smoking	No	669	14.2	0.7 (0.4–1.4)	79.2	1.0	41.3	1.0
	Yes	65	18.5	1.0	67.7	0.6 (0.3–1.0)	44.6	1.1 (0.7–1.9)
OHI–S (Soft deposit)	<1 (Good)	179	50.3	126.4 * (17.3–924.2)	45.3	1.0	18.4	1.0
	1– (Fair)	429	3.7	4.8 (0.6–36.9)	91.8	13.6 * (8.7–21.4)	45.2	3.7 * (2.4–5.6)
	2+ (Poor)	126	0.8	1.0	78.6	4.4 * (2.6–7.4)	61.9	7.2 * (4.3–12.1)

◈ 16 males and 11 females with missing mothers’ education; ⁂ 15 males and 26 females with missing fathers’ education.
